# CD47 Expression Predicts Unfavorable Prognosis in Clear Cell Renal Cell Carcinoma after Curative Resection

**DOI:** 10.3390/diagnostics12102291

**Published:** 2022-09-22

**Authors:** Hosub Park, Seungyun Jee, Seongsik Bang, Hwangkyu Son, Hyebin Cha, Jaekyung Myung, Jongmin Sim, Yeseul Kim, Seungsam Paik, Hyunsung Kim

**Affiliations:** 1 Department of Pathology, Seoul Hospital, Hanyang University College of Medicine, Seoul 04763, Korea; 2 Department of Pathology, Anam Hospital, Korea University College of Medicine, Seoul 02841, Korea

**Keywords:** clear cell renal cell carcinoma, CD47, immunohistochemistry, prognosis

## Abstract

The role of CD47 expression as a ‘do not eat me’ signal that inhibits phagocytosis of tumor cells by macrophages is well established. Immune checkpoint therapy that targets CD47 has been successful in preclinical trials and is currently undergoing clinical investigation for various human malignancies. Here, the clinicopathological correlation with CD47 expression in clear cell renal cell carcinoma (ccRCC) was explored. CD47 expression was evaluated by immunohistochemical staining in tissue microarray sections of 235 ccRCC tissues. CD47 expression was observed in 28 (11.9%) of 235 ccRCC tissues and was significantly associated with higher WHO/ISUP grade (*p* = 0.001), frequent lymphovascular invasion (*p* = 0.036), frequent renal vein thrombus (*p* = 0.018), frequent sinus fat invasion (*p* = 0.004), frequent sarcomatous change (*p* = 0.001), higher pT stage (*p* = 0.002), higher pN stage (*p* = 0.002), higher pM stage (*p* < 0.001), and advanced American Joint Committee on Cancer stage (*p* = 0.002). In the survival analyses, positive CD47 expression was associated with cancer-specific survival (*p* = 0.003). However, positive CD47 expression was not associated with recurrence-free survival. In conclusion, CD47 expression was associated with adverse clinicopathological parameters and cancer-specific survival in patients with ccRCC.

## 1. Introduction

Renal cell carcinoma (RCC) is the most common cancer of the kidney and represents 2% of cancer diagnoses and deaths worldwide [1]. RCC ranks 6th in men and 10th in women among cancers globally, and it is estimated to be the 9th most common cancer in both sexes in Korea [2]. Although rates are low, the incidence has doubled in developed countries over the past half century [3]. Despite the improvement in diagnosis and therapeutic approaches for RCC during the recent two decades, it remains one of the most lethal malignancies of the urinary system [4].

Clear cell renal cell carcinoma (ccRCC) is the most common histologic subtype of RCC and accounts for more than 80% of all cases [5]. Most available treatments focus on ccRCC and RCCs with non-clear cell histologic subtypes do not have a similarly refined treatment paradigm [6]. Partial or radical nephrectomy is the first-line treatment for localized ccRCC, and systemic therapy is a recommended treatment option for advanced ccRCC or metastatic ccRCC [7,8]. The role of adjuvant systemic therapy for patients with stage I to III has been limited, although the U.S. Food and Drug Administration has approved adjuvant therapy for localized ccRCC [7]. Broadly, current systemic treatment for ccRCC is divided into targeted therapies and immunotherapies [9]. The first agent approved in the targeted therapy category was sorafenib, which targets vascular endothelial growth factor receptor (VEGFR), rapidly accelerated fibrosarcoma, and other growth factors [6]. Subsequently, similar agents including sunitinib, pazopanib, axitinib, and bevacizumab were approved [10]. Currently available immunotherapies include agents targeting the cancer immune system via inhibition of immune evasion or increase in T-lymphocyte priming [9]. Three programmed cell death protein 1 and programmed death-ligand 1 inhibitors (nivolumab, pembrolizumab, and avelumab) and one cytotoxic T-lymphocyte–associated protein 4 inhibitor (ipilimumab) have been approved [7]. However, ccRCC patients show an inconsistent prognosis and limited responses to the aforementioned treatments [11]. Therefore, the discovery of a new biomarker that better predicts ccRCC prognosis and can be a candidate for a therapeutic target is needed.

CD47 is a transmembrane protein that is ubiquitously expressed on the cell surface and sends a “do not eat me” signal to phagocytes, acting as an immune checkpoint [12]. CD47 initiates the signaling cascade by interacting with signal regulatory protein-α (SIRPα) expressed on macrophages [13]. This interaction activates tyrosine phosphatases SHP-1 and SHP-2 and ultimately prevents engulfment, potentially by inhibiting the accumulation of myosin-IIA at the cell membrane [13,14]. CD47 overexpression has been reported in various human malignancies and generally heralds poor prognosis [15]. As a therapeutic target, anti-CD47 antibody has shown promising results in diffuse large B-cell lymphoma, pediatric malignant brain tumor, ovarian cancer, hepatocellular carcinoma, and lung cancer [16,17,18,19,20]. Immune checkpoint agents targeting CD47 have demonstrated success in preclinical investigations and are now under clinical trials for various types of human malignancies [21]. One anti-CD47 antibody, magrolimab, is at the forefront of clinical development, and its combination treatment has been well tolerated in phase Ib/II studies with encouraging response rates [22]. However, CD47 expression in ccRCC has been rarely reported and its correlation with various clinicopathological parameters has not been well characterized [23].

To reveal the clinicopathological significance of CD47 expression in human ccRCC, CD47 protein expression was evaluated in 235 ccRCC tissue samples. Furthermore, to reveal the prognostic role of CD47 expression, survival analyses were performed.

## 2. Materials and Methods

### 2.1. Patients and Tumor Samples

A total of 241 patients diagnosed with ccRCC and who received partial or radical nephrectomy at Hanyang University Hospital (Seoul, Korea) between 2001 and 2017 was enrolled in this cohort. Patients without qualified tissue paraffin blocks were excluded, finally leaving 235 patients. Medical records and hematoxylin and eosin (H&E)-stained slides were reviewed. The 8th AJCC TNM staging system and a protocol for examining specimens from patients with invasive carcinoma of renal tubular origin (College of American Pathologists) were used to determine the pathological staging and other pathological characteristics [24,25]. Pathological parameters included tumor size, World Health Organization/International Society of Urological Pathology (WHO/ISUP) grade, lymphovascular invasion, renal vein tumor thrombus, sinus fat invasion, perirenal soft tissue invasion, tumor necrosis, sarcomatoid change, pathological T (pT) stage, pathological N (pN) stage, pathological M (pM) stage, and American Joint Committee on Cancer (AJCC) stage. Cancer-specific survival (CSS) was defined as the duration from as the date of surgery until the date of death due to the ccRCC. Recurrence-free survival (RFS) was defined as the duration from the date of surgery until the date of disease progression, relapse, or death from ccRCC.

### 2.2. Tissue Microarray (TMA) Construction

A manual tissue microarrayer (Unitma, Seoul, Korea) was used to construct TMAs from archival formalin-fixed, paraffin-embedded tissue blocks. The H&E-stained slides were reviewed to select the most representative area of carcinoma without necrosis and hemorrhage. A 2 mm tissue cylinder was punched from the previously marked area on each donor paraffin block and transplanted into the recipient block (Unitma). Each TMA comprised 5 × 6 samples.

### 2.3. Immunohistochemical Staining

CD47 immunohistochemical staining was performed with 4 µm thick sectioned slides from TMA blocks. The Ventana BenchMark XT autostainer (Ventana Medical Systems, Tucson, AZ, USA) was used according to the manufacturer’s protocol. Anti-CD47 monoclonal antibody (1:200 dilution, EPR21794; Abcam, Cambridge, UK) was used to detect CD47 expression.

### 2.4. Interpretation of Immunohistochemical Staining

**Figure 1:**
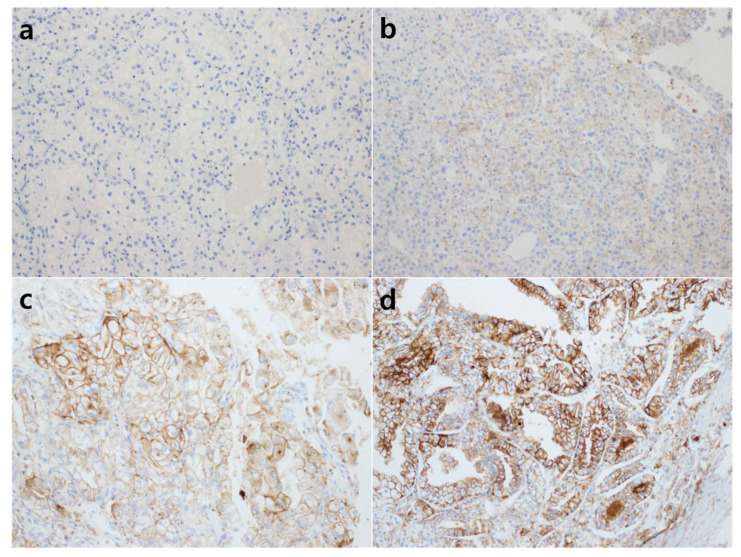
Representative microphotographs of CD47 immunohistochemical staining. The intensity of nuclear staining was determined as 0: negative (**a**); 1+: weak (**b**); 2+: moderate (**c**); 3+: strong (**d**) ((**a**–**d**), original magnification ×200).

The tumor cells with membranous staining was evaluated by the H-score method. The staining intensity was scored by a 4-tier system (0: negative, 1+: weak, 2+: moderate, 3+: strong), and the percentage of stained tumor cells was evaluated. Figure 1 shows representative microphotographs stratified by staining intensity. The H-score was determined by the following calculation method: H-score = % of weakly stained cells (1+) + 2 × % of moderately stained cells (2+) + 3 × % of strongly stained cells (3+). Positive CD47 expression was defined as H-score ≥ 5 (≥5% tumor cell membrane staining) according to reference studies [26,27]. All immunohistochemical assessments were blinded to the clinicopathological parameters and patient survival.

### 2.5. Statistical Analysis

Statistical analysis was performed with SPSS software version 21 (IBM Corp., Armonk, NY, USA). The correlation between CD47 expression and clinicopathological factors including sex, tumor size, WHO/ISUP grade, lymphovascular invasion, renal vein tumor thrombus, sinus fat invasion, perirenal soft tissue invasion, tumor necrosis, sarcomatoid change, pT stage, pN stage, pM stage, and AJCC stage was assessed using the chi-square test. The survival analyses were performed using the Kaplan–Meier method with a log-rank test and the Cox proportional hazard regression model. A *p*-value less than 0.05 was considered as statistically significant.

## 3. Results

### 3.1. Patient Characteristics

The patient characteristics are summarized in Table 1. The median patient age was 57 years (13–84 years), and the male to female ratio was 2.3:1. According to pathological evaluation, the mean tumor size was 4.14 cm (±2.69). In histologic grade, 25 cases (10.6%) were WHO/ISUP grade 1, 118 (50.2%) were WHO/ISUP grade 2, 75 (31.9%) were WHO/ISUP grade 3, and 17 (7.2%) were WHO/ISUP grade 4. In the AJCC staging system, 170 cases (72.3%) were pT1, 10 (4.3%) were pT2, 52 (22.1%) were pT3, and 3 (1.3%) were pT4.

### 3.2. Correlations between CD47 Expression and Clinicopathological Parameters

Positive CD47 expression was observed in 28 (11.9%) of 235 ccRCC tissues. Positive CD47 expression was significantly associated with higher WHO/ISUP histologic grade (*p* = 0.001), frequent lymphovascular invasion (*p* = 0.036), frequent renal vein thrombus (*p* = 0.018), frequent sinus fat invasion (*p* = 0.004), frequent sarcomatoid change (*p* = 0.001), higher pT stage (*p* = 0.002), higher pN stage (*p* = 0.002), higher pM stage (*p* < 0.001), and higher AJCC stage (*p* = 0.002) (Table 2).

### 3.3. Prognostic Role of CD47 Expression in ccRCC

**Figure 2:**
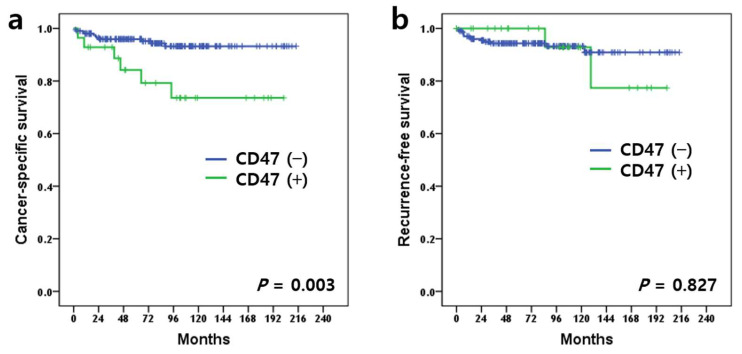
Kaplan–Meier survival curves stratified by CD47 expression. Cancer-specific survival (**a**) (*p* = 0.003, log-rank test) and recurrence-free survival (**b**) (*p* = 0.827, log-rank test).

Of the total 235 patients, 18 died of ccRCC and 15 experienced cancer recurrence. Patients with positive CD47 expression had a poorer prognosis for cancer-specific survival (CSS) than patients with negative CD47 expression (*p* = 0.003) (Figure 2a). However, there was no significant difference between the positive and negative CD47 expression groups for recurrence-free survival (*p* = 0.827) (Figure 2b). Univariable Cox regression analysis demonstrated that positive CD47 expression was a poor prognostic factor (*p* = 0.006) along with other known pathological parameters of WHO/ISUP grade, lymphovascular invasion, sinus fat invasion, sarcomatoid invasion, and pathologic TNM stage in CSS (Table 3). However, positive CD47 expression was not an independent prognostic factor in the multivariable analysis (Table 3).

## 4. Discussion

CD47, a highly glycosylated protein, normally acts as a “do not eat me” signal in healthy cells to prevent phagocytosis by macrophages, whereas CD47 is downregulated in redundant cells, promoting macrophages to clear them [12]. CD47 is also overexpressed in various types of cancer cells to avoid the innate immune system [12]. CD47 overexpression has been reported in various human malignancies including non-Hodgkin lymphoma, stomach cancer, oral squamous cell carcinoma, lung cancer, breast cancer, and hepatocellular carcinoma and has been correlated with adverse prognosis [28,29,30,31,32,33]. After the discovery of its role in tumor evasion of immune surveillance, CD47 has become a novel biomarker for cancer immunotherapy, and several anti-CD47 antibodies have been studied, including B6H12, Hu5F9-G4, and ZF1 [34,35,36]. Immune checkpoint agents targeting CD47 have demonstrated success in preclinical investigations and are now under clinical trials [21]. However, CD47, as a prognostic biomarker and potential therapeutic target, lacks research focusing on its significance in ccRCC.

To date, there are two studies investigating CD47 expression in RCC and ccRCC, a subtype of RCC. In Gazel et al., the intensity of CD47 immunohistochemical staining in RCC was analyzed and correlated with histopathological features [37]. A significant correlation was not found between CD47 expression and tumor size, capsular invasion, vascular invasion, or distant metastasis [37]. However, strong CD47 expression was frequently identified in patient groups with non-clear cell RCC subtypes, higher Fuhrman nuclear grades, lymph node metastasis, and advanced stages [37]. We additionally subdivided the patients into higher and lower CD47 expression using intensity scores. Higher CD47 expression tends to correlate with aggressive phenotypes; however, the number of higher expression group was too small to be statistically significant. Establishing a standard cut-off for CD47 expression requires additional studies using different cut-off values and validation studies in clinical trials. Jiang et al. explored the significance of CD47 expression in ccRCC and correlated it with CD8+ T lymphocyte infiltration, molecular features, and response to combination therapy [23]. The patient group with high CD47 expression was prone to dismal prognosis in both overall survival and recurrence-free survival and was more likely to be classified as ccB molecular subtype [23]. Additionally, high CD47 expression correlated with an immunosuppressed tumor microenvironment of exhausted CD8+ T cells [23]. Interestingly, high CD47 expression was associated with improved response to VEGFR tyrosine kinase inhibitor + immune checkpoint inhibitor combination therapies, and the authors suggested CD47 as a potential target for immunotherapy in ccRCC [23].

In this study, positive CD47 expression was identified in a minority (11.9%) of ccRCC samples. It correlated with aggressive pathological phenotypes, including higher histologic grade, the presence of lymphovascular invasion, renal vein thrombus, sinus fat invasion, frequent sarcomatoid change, higher pathological stages, and poor patient survival. These findings suggest that CD47 protein expression in ccRCC demonstrates an adverse role in tumor immunity and prognosis, consistent with the results from previous studies. The aggressive phenotypes attributes to the fact that CD47 promotes tumor cell growth and motility [38,39]. A preclinical study demonstrated that the downregulation of CD47 inhibits the growth and metastasis of human cancer cells [40]. Recent clinical data revealed that anti-CD47 antibody induced encouraging anti-cancer effects, suggesting that CD47 is also a potential therapeutic target in ccRCC [22]. However, several limitations exist in this study. First, we performed a retrospective analysis of limited ethnicity (mostly Korean) in a single medical center. Second, a detailed mechanism of CD47 expression involving tumor immune evasion in ccRCC has not been mentioned. We also applied only a single marker, which requires other biomarkers relevant to the tumor immune environment, such as macrophages and anti-tumor T cells [41]. Further molecular study is needed to identify the cancer-specific immune evasion mechanism of CD47 in ccRCC. Third, the therapeutic benefit of CD47 expression according to type of adjuvant therapy has not been determined because patients enrolled in this study did not receive immunotherapy or targeted therapy. A large-scale cohort study that can predict response to current treatment according to CD47 expression is needed.

## 5. Conclusions

CD47 expression was investigated in 235 ccRCC patients. A positive CD47 expression was identified in 11.9% of ccRCC tissues and was significantly correlated with aggressive phenotypes and poor patient survival. The expression of CD47 in ccRCC is deemed a valuable therapeutic target for immunotherapy.

## Figures and Tables

**Table 1:** Baseline characteristics of the patients (*n* = 235).

Factors	Value (%)
Number of patients	235 (100%)
Median age at surgery (years)	57 (±12.77)
Mean tumor size (cm)	4.14 (±2.69)
Sex	
Male	168 (69.7%)
Female	73 (30.3%)
WHO/ISUP grade	
Grade 1	25 (10.6%)
Grade 2	118 (50.2%)
Grade 3	75 (31.9%)
Grade 4	17 (7.2%)
pT stage	
pT1	170 (72.3%)
pT2	10 (4.3%)
pT3	52 (22.1%)
pT4	3 (1.3%)

WHO/ISUP: World Health Organization/International Society of Urological Pathology.

**Table 2:** Correlation between CD47 expression and clinicopathologic factors in clear cell renal cell carcinoma.

Clinicopathologic Factors	*n*	CD47 Expression		*p*-Value
		Negative (%) (*n* = 207)	Positive (%) (*n* = 28)	
Sex				0.465
Male	165	147 (89.1%)	18 (10.9%)	
Female	70	60 (85.7%)	10 (14.3%)	
Tumor size				0.062
≤4 cm	139	127 (91.4%)	12 (8.6%)	
>4 cm	96	80 (83.3%)	16 (16.7%)	
WHO/ISUP grade				0.001
Grade 1 & 2	143	134 (93.7%)	9 (6.3%)	
Grade 3 & 4	92	73 (79.3%)	19 (20.7%)	
Lymphovascular invasion *				0.036
Absent	193	174 (90.2%)	19 (9.8%)	
Present	42	33 (78.6%)	9 (21.4%)	
Renal vein thrombus				0.018
Absent	202	182 (90.1%)	20 (9.9%)	
Present	33	25 (75.8%)	8 (24.2%)	
Sinus fat invasion				0.004
Absent	212	191 (90.1%)	21 (9.9%)	
Present	23	16 (69.6%)	7 (30.4%)	
Perirenal soft tissue invasion				0.437
Absent	204	181 (88.7%)	23 (11.3%)	
Present	31	26 (83.9%)	5 (16.1%)	
Tumor necrosis				0.130
Absent	199	178 (89.4%)	21 (10.6%)	
Present	36	29 (80.6%)	7 (19.4%)	
Sarcomatoid change				0.001
Absent	220	198 (90.0%)	22 (10.0%)	
Present	15	9 (60.0%)	6 (40.0%)	
pT stage				0.002
pT1 & pT2	180	163 (90.6%)	17 (9.4%)	
pT3 & pT4	55	44 (80.0%)	11 (20.0%)	
pN stage				0.002
pN0	231	206 (89.2%)	25 (10.8%)	
pN1	4	1 (25.0%)	3 (75.0%)	
pM stage				<0.001
pM0	228	205 (89.9%)	23 (10.1%)	
pM1	7	2 (28.6%)	5 (71.4%)	
AJCC stage				0.002
I, II	179	163 (91.1%)	16 (8.9%)	
III, IV	56	44 (78.6%)	12 (21.4%)	

WHO/ISUP: World Health Organization/International Society of Urological Pathology; AJCC: American Joint Committee on Cancer. * Renal vein thrombus included.

**Table 3:** Univariable and multivariable Cox regression analysis of pathological parameters for cancer-specific survival in clear cell renal cell carcinoma. (*n* = 235).

Factors	Univariable Analysis		Multivariable Analysis	
	HR (95% CI)	*p*-Value	HR (95% CI)	*p*-Value
CD47 expression (negative vs. present)	3.985 (1.473–10.785)	0.006	2.264 (0.563–9.099)	0.250
WHO/ISUP grade (grade 1, 2 vs. grade 3, 4)	9.584 (2.769–33.169)	<0.001	2.624 (0.493–13.982)	0.258
Lymphovascular invasion (absent vs. present)	93.311 (12.410–701.613)	<0.001	16.946 (0.909–315.757)	0.058
Sinus fat invasion (absent vs. present)	6.494 (2.516–16.759)	<0.001	1.883 (0.546–6.493)	0.316
Sarcomatoid change (absent vs. present)	18.428 (7.177–47.317)	<0.001	1.144 (0.320–4.086)	0.836
pT stage (pT1, 2 vs. pT3, 4)	65.998 (8.776–496.315)	<0.001	4.236 (0.218–82.162)	0.340
pN stage (pN0 vs. pN1)	19.395 (5.557–67.987)	<0.001	6.110 (1.016–36.757)	0.048
pM stage (pM0 vs. pM1)	63.622 (20.985–192.980)	<0.001	13.177 (2.405–72.206)	0.003

## Data Availability

Not applicable.
